# Genomic factors underlying sex differences in trauma-related disorders

**DOI:** 10.1016/j.ynstr.2021.100330

**Published:** 2021-04-23

**Authors:** Olga Y. Ponomareva, Kerry J. Ressler

**Affiliations:** a Neuropsychiatry Translational Research Fellowship Program, Boston VA Healthcare System, Boston, MA, USA; b McLean Hospital, Harvard Medical School, Belmont, MA, USA

**Keywords:** PTSD, Sex differences, Genetic, Genome wide association study, GWAS, Epigenetic, RNA, Transcriptomic, Sexual dimorphism, Trauma

## Abstract

Post-traumatic stress disorder (PTSD) is a devastating illness with treatment that is effective in only approximately half of the population. This limited rate of response highlights the necessity for research into underlying individual biological mechanisms that mediate development and progression of this disease, allowing for identification of patient-specific treatments. PTSD has clear sex differences in both risk and symptom patterns. Thus, one approach is to characterize trauma-related changes between men and women who exhibit differences in treatment efficacy and response to trauma. Recent technological advances in sequencing have identified several genomic loci and transcriptional changes that are associated with post-trauma symptomatology. However, although the diagnosis of PTSD is more prevalent in women, the genetic factors underlying sex differences remain poorly understood. Here, we review recent work that highlights current understanding and limitations in the field of sex differences in PTSD and related symptomatology.

## 1 Introduction

Posttraumatic Stress Disorder (PTSD) is a complex mental illness with a high lifetime prevalence. It is often accompanied by significant comorbid syndromes including depression, substance abuse, and elevated suicide risk. According to the National Comorbidity Survey Replication, a large-scale assessment of the epidemiology of mental illness in the United States conducted between February 2001 and April 2003, the lifetime prevalence of PTSD in adult Americans was 6.8% (Kessler et al., 2005), with women having almost twice the likelihood of men to have a PTSD diagnosis. As a psychiatric illness, PTSD is unique in that it requires an environmental stressor to fulfill criteria for a diagnosis. This stressor must be of a traumatic nature and can be of direct or indirect exposure. Other diagnostic criteria include persistent reexperiencing of this stressor, avoidance of reminders of the trauma, negative thoughts or feelings associated with the event, and heightened reactivity to triggers that serve as reminders of the trauma (Diagnostic and Statistical Manual of Mental Disorders, 5th edition). Interestingly, men and women show a different response to treatments commonly used for PTSD, with women having a more robust response to trauma-focused therapy (Wade et al., 2016). Antidepressants in the class of Selective Serotonin Reuptake Inhibitors (SSRIs) are the first-line pharmacological treatment for PTSD and major depressive disorder (MDD). Several recent studies suggest a differential response to this medication based on sex, further implying a sex difference in the underlying molecular mechanisms of these disorders (Sramek et al., 2016; Khan et al., 2005; LeGates et al., 2019).

Sex differences in PTSD prevalence are not fully explained by the type of incident trauma, although women appear to be more vulnerable to experiencing sexual trauma, such as rape, molestation and physical abuse, while men are more likely to experience events related to combat, threat with a weapon, or being a witness to trauma (Kessler, 1995). When controlling for the type of traumatic event experienced, most studies suggest that sex differences in the prevalence of PTSD remain; however, one study of intimate partner violence suggested that sex differences resolve after controlling for type of trauma, and history of sexual victimization, rather than sex itself, is the driving factor in the observed difference in PTSD prevalence (Tolin and Foa, 2006; Breslau and Anthony, 2007; Cortina and Kubiak, 2006). Although questions about the type and extent of environmental exposure as a contribution to sex differences in PTSD remain, results from several recent large-scale genomic studies suggest that women of both European and African ancestry have significantly higher PTSD heritability than men. These data provide additional strong support for a role of genetic factors interacting with sex differences in the development of this disorder (Duncan et al., 2018; Nievergelt et al., 2019).

Despite emerging evidence of the role of sex on development of trauma-related disorders and on treatment, few human studies have examined the role of genetic factors in this dichotomy. More literature is available from studies of transcriptional changes in depressive disorders and in pre-clinical work with rodents, which suggest a sex difference in pathways involved in immune function and neuronal structure (Brivio et al., 2020). Work conducted in postmortem brain tissue of subjects with major depressive disorder (MDD) suggests limited overlap in the transcriptional profiles between males and females, suggesting differences in underlying molecular mechanisms following stress exposure (Labonté et al., 2017; Seney et al., 2018).

**Fig. 1:**
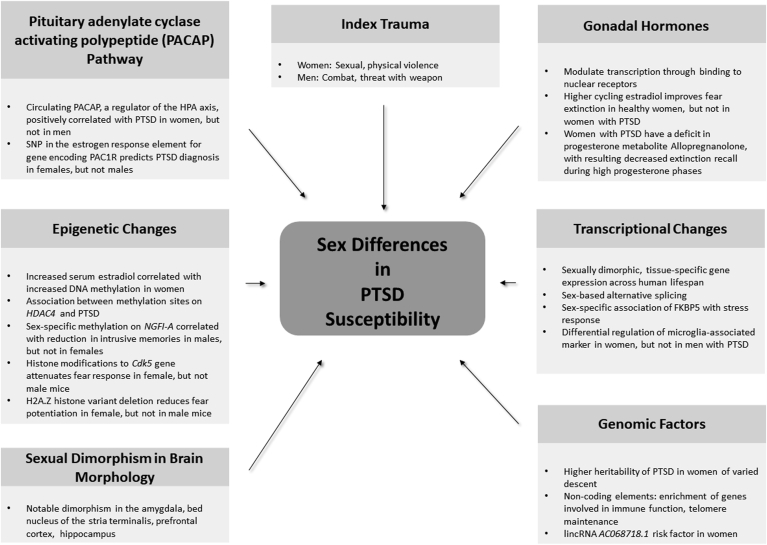
Notable contributions to sex differences in development of PTSD.

These observations have led to increased focus on sex as a risk factor for PTSD, with recent findings of the initial response to trauma, role of neurosteroids, environmental and genetic factors (Irish et al., 2011; Christiansen and Hansen, 2015; Goldstein et al., 2010; Cruz et al., 2019; Christiansen and Berke, 2020). Furthermore, several sexually dimorphic brain regions involved in PTSD have been identified, including the amygdala, bed nucleus of the stria terminalis (BNST), prefrontal cortex, and the hippocampus (Oliva et al., 2020; Hines et al., 1992). Multiple recent studies have also highlighted changes in gene expression between males and females; however, limited work has been done on the role of sex differences on gene expression in PTSD (Gegenhuber and Tollkuhn, 2020; Xu et al., 2014; Naqvi et al., 2019). In this review, we will further explore the above observations and provide an overview of current literature of genomic and transcriptional factors underlying sex differences in PTSD. A summary of notable contributions to sex differences in PTSD are illustrated in Fig. 1.

## 2 Role of genetic factors in PTSD-Related sex differences

Genome wide association studies (GWAS) are a useful tool for the study of genetic variables with inherent differences in common DNA sequence variants (single nucleotide polymorphisms, SNPs) that may contribute to disease pathology. GWAS scan the entire genome for associations of genetic elements (SNPs) with a trait of interest. Large-scale GWAS have begun to elucidate the correlations of genetic variants with PTSD symptomatology, although few studies have reported the results stratified by sex (Daskalakis et al., 2018).

Recent GWAS conducted in military veterans with PTSD identified several loci, including those corresponding to genes in the corticosteroid and steroid function, however, they did not report effects stratified by sex (Gelernter et al., 2019; Stein et al., 2019a). In a GWAS conducted in mainly African American women and replicated in women of European descent, Guffanti et al. identified an enrichment of genes involved in immune function, telomere maintenance, and a novel lincRNA gene as a risk factor for PTSD (Guffanti et al., 2013). The results suggest a potential role of non-coding elements in the development of PTSD in women. Another large, multiethnic GWAS from the Psychiatric Genomics Consortia (PGC) found that heritability of PTSD for women of European-American ancestry was substantially higher than for males; however, the study found no SNPs that reached genome-wide significance (Duncan et al., 2018). A follow-up PGC study of a larger population, including subjects of European and African descent, found three genome-wide significant loci. When stratified by sex, it implicated three other genome-wide significant loci in men, but not in women (Nievergelt et al., 2019). Additionally, our preliminary (unpublished) work with GWAS stratified by sex has suggested that the patterns of significant loci in PTSD appear different and at different chromosomal locations in men and women, suggesting differential sex-dependent genetic pathways mediating stress-related disorders.

Although the field of PTSD has only recently started to gain insights from large-scale GWAS, studies that include stratification by sex suggest underlying genetic differences may contribute to variability of PTSD prevalence in males and females. Of note, many sexually dimorphic genes in the human genome are located on X and Y chromosomes, which have not been included in most GWAS analyses to date. Insights from human studies could also be complemented with work in animal models; however, recent rodent GWAS and large-scale transcription studies exploring changes in gene-expression in circuitry related to PTSD-symptomatology included only male animals (Krishnan et al., 2007; Gray et al., 2014; Muhie et al., 2015).

## 3 Role of differential transcription in sex differences in PTSD

A growing body of literature suggests sexually dimorphic gene expression across a wide variety of model organisms. Approximately half of expressed genes in the fruitfly *Drosophila* show sexual dimorphism, and sexually dimorphic genes are involved in the majority of changes in adult gene expression during recent evolution (Ranz et al., 2003). Using tissue from adult mice, Yang et al. found sexual dimorphism in the expression of approximately 10,000 genes in somatic tissues, with 13.6% change in active gene expression in the brain (Yang et al., 2006). Notably, the authors found a sex-chromosome enrichment of sexually-dimorphic genes, which has also been observed in human studies (Yang et al., 2006; Vawter et al., 2004). Yang et al. note that some of the sex differences observed in tissue from the whole mouse brain may have been masked by the heterogeneity of different brain regions, highlighting importance of segregation by brain regions in further analyses.

In humans, gene expression remains sexually dimorphic across the lifespan, with sex-specific effects in gene expression related to brain aging (Berchtold et al., 2008). In human postmortem brain tissue, approximately 2.5% of expressed genes in the brain have sexually dimorphic expression in healthy individuals (North American Brain Expression ConsortiumTrabzuni et al., 2013). Interestingly, over 80% of these genes exhibit sexually dimorphic changes due to sex-biased alternative splicing. Several of the brain regions examined, including the hippocampus and the temporal cortex, exhibited sexually dimorphic gene expression that localized primarily to the X and Y chromosomes. Unfortunately, as mentioned above due to alignment complexity, the sex chromosomes are not often examined in current large-scale GWAS studies (North American Brain Expression ConsortiumTrabzuni et al., 2013).

To address the role of differential gene expression in PTSD, several studies have begun to utilize postmortem human brain tissue; however, to date, there are a limited number of Brain Banks that include tissue from PTSD subjects. These include Lieber Institute for Brain Development, McLean Hospital Harvard Brain Tissue Resource Center, the Leahy-Friedman National PTSD Brain Bank, and the Netherlands Brain Bank (de Lange, 2017; Friedman et al., 2017; Mighdoll et al., 2018). Thus, the number of such studies and their power to discriminate effects of sex differences, remains small.

In one small study that included mostly female subjects, Su et al. identified differential expression of genes in the mitochondrial networks and oxidative phosphorylation using a mitochondrial-focused microarray in the dorsolateral prefrontal cortex (Su et al., 2008). A limitation of this study is that it included mostly female subjects and examined a limited number (n = 6) of tissues from patients with PTSD. A follow up study in a rodent model found further evidence of dysregulation of mitochondrial genes after tail shock stress, however, only male animals were included in this analysis (Zhang et al., 2015).

A better powered recent publication provided the largest transcriptomic study of human brain tissue to date, with a transcriptome-wide analysis of 143 subjects from four subregions of the prefrontal cortex in males and females (Girgenti et al., 2020). The results suggest marked sex-specific differences in the transcriptome of subjects with PTSD, and were observed in all tissue regions examined. Compared with healthy controls, female subjects with PTSD had more differentially expressed genes than male subjects. The study identified several key drivers in combined-sex as well as in female and male-specific networks, including *ELFN1*, an allosteric modulator of metabotropic glutamate receptors, and multiple GABA-related transcripts that are downregulated in the same network, suggesting decreased GABA signaling in PTSD. The study also found a previously-identified mediator of PTSD symptomatology, FK506-Binding Protein 5 (*FKBP5),* as upregulated in the dorsolateral prefrontal cortex of females with PTSD.

FKBP5 is a chaperone protein for the glucocorticoid receptor (GR) that has been implicated in the pathophysiology of PTSD, with several identified SNPs exhibiting a gene × environment interaction with PTSD symptoms (Binder et al., 2008; Holmes et al., 2017). When bound to glucocorticoid receptor, FKBP5 prevents its translocation to the nucleus, thus impairing GR-mediated downstream gene expression. A prior study using postmortem tissue from mostly male subjects identified a correlation between mRNA levels of *FKBP5* and dendritic spine morphology (Young et al., 2015). These results suggest that FKBP5 may act as an upstream regulator of a signaling cascade involved in development of PTSD symptomatology. It also may act as a key player in immune system dysregulation, which is thought to play a role in the development of PTSD symptomatology (Michopoulos et al., 2017). In addition to its role in regulation of glucocorticoid receptor activity, FKBP5 interacts with progesterone receptors, and may promote activity of the estrogen receptor (Hubler et al., 2003; Shrestha et al., 2015). Recent work suggests that the efficacy of an FKBP5 inhibitor, SAFit2, on mediation of anxiety-like behaviors in rats withdrawing from cocaine exposure, is dependent on the specific stage of the rodent estrous cycle, further implicating the role of gonadal hormones in FKBP5 signaling (Connelly et al., 2020). Work in healthy human subjects has also demonstrated a sex-specific effect, with mRNA levels of *FKBP5* collected from blood correlating with scores on depression and anxiety rating scales in women, but not in men (Lee et al., 2018). The same study found a sex-specific correlation of *FKBP5* mRNA levels with bedtime cortisol levels in female, but not in male subjects. Taken together, this work suggests that FKBP5 may be a useful sex-specific biomarker of stress response and subsequent development of PTSD, and further elucidation of this signaling pathway may yield insights into genetic mechanisms that regulate sexual dimorphism of PTSD symptomatology.

Another recent study using postmortem brain tissue provides evidence for a differential regulation of microglia-associated marker in the orbitofrontal cortex of women with PTSD – an effect that was not observed in tissue collected from male subjects (Bhatt et al., 2020). Interestingly, the same effect was not found in the subgenual prefrontal cortex, highlighting tissue-specificity of sexual dichotomy in gene expression (Bhatt et al., 2020).

In the absence or readily-available brain tissue for genetic studies, peripheral blood biomarkers have been used to study the contribution of inflammatory markers to PTSD development, including impaired glucocorticoid signaling; however, most of these studies have focused on male subjects (Breen et al., 2015). One study that examined differential gene expression from monocytes of male and female subjects found sex-specific differential regulation of cAMP response element binding protein (CREB) and activating transcription factor (ATF) in subjects with PTSD, although the study was limited by a small number of female subjects (O'Donovan et al., 2011).

To further overcome limitations of human postmortem tissue availability in the study of PTSD transcriptomics, established rodent models of PTSD symptomatology can be used as a powerful approach to explore transcriptional changes following stress and trauma exposure. Two recent transcriptomic analyses in rodents examined sex differences in fear-related circuitry. One group used a previously-published dataset of male and female rats exposed to predator-scent stress. They then separated animals into resilient or vulnerable behavioral response groups, to explore sex-specific cell composition and transcriptome differences (Daskalakis et al., 2014). They found that female animals showed a greater variability in cellular composition in the amydgala (Kim and Uddin, 2020). Females with an enhanced response to the stressor also demonstrated changes in synaptic plasticity in the hippocampus, with upregulation of TNFα signaling. Focusing on the amygdala, male rats that exhibited an extreme behavioral response to a stressor had decreased expression of neuropeptide activity, including cholecystokinin, neuropeptide Y, and vasoactive intestinal peptide, while females exhibited changes in dopamine D_2_ receptor signaling and glucose homeostasis. In the periphery, blood markers of inflammation were upregulated in the resilient group, whereas a sexually dimorphic dysregulation of inflammatory pathways was found in animals more susceptible to stress (Kim and Uddin, 2020).

Several studies have begun to explore sex differences in immune dysregulation in PTSD. To follow up on a possible association of corticotropin-releasing hormone (CRH) receptor variants identified in prior GWAS studies, recent work by McCullough and colleagues examined transcription in CRH-expressing cells in the central amygdala of male and female mice after fear conditioning and extinction (McCullough et al., 2020). In male mice, upstream regulator analysis identified a role for CREB as critical regulator of gene expression pathways that were suppressed during extinction. Female mice had changes in expression that were highly correlated to the changes observed in male mice; however, no differentially expressed genes surviving false discovery rate were found in females, suggesting more variance in their transcriptional profiles (McCullough et al., 2020). Another rodent study using predator-scent-stress exposure to explore genome-wide transcription changes in blood, amygdala, and hippocampus of male and female rats identified glucocorticoid signaling as the only converging pathway across tissue and sex (Daskalakis et al., 2014). A different number of genes were associated with vulnerability or resilience to stress in males and females across the tissues examined, further highlighting differential regulation of pathways involved in stress response (Daskalakis et al., 2014; Stein et al., 2019b).

## 4 Role of gonadal hormone fluctuations on transcriptional regulation in PTSD

Circulating gonadal hormone levels regulate response to environmental stressors, with multiple studies demonstrating modulatory effects of estrogen, testosterone, and progesterone (Glover et al., 2013; Domonkos et al., 2018). Note that a primary mechanism of actions of these hormones is through their binding to nuclear receptors that function as transcriptional modulators of gene expression. Thus, one mechanism by which sex differences in RNA transcription can be established is through changing levels of circulating hormones that signal via hormonal-response elements.

Perhaps the best-characterized example of one such pathway in the stress response is signaling through pituitary adenylate cyclase-activating polypeptide (PACAP), a member of the secretin/vasoactive intestinal polypeptide family. Together with its receptor, PAC1R, PACAP is expressed in brain regions involved in stress response, and shows an increase in expression after acute and chronic stress exposure (Shioda et al., 1997; Kozicz and Arimura, 2002; Palkovits et al., 1995; Arimura et al., 1991; Hammack et al., 2009; Johnson et al., 2020). In human and rodent studies, the effect of PACAPergic signaling on stress response is sexually dimorphic (Ramikie and Ressler, 2016). Circulating levels of a biologically active PACAP peptide, PACAP38, were positively correlated with PTSD diagnosis and symptom clusters specifically in female, but not in male, subjects (Ressler et al., 2011; Miyata et al., 1990).

PACAP is a known paracrine mediator of the hypothalamic-pituitary-adrenal (HPA) axis (Nussdorfer and Malendowicz, 1998), and also regulates levels of activity-dependent neuroprotective protein (ADNP) (Zusev and Gozes, 2004), which predisposes rodents to a sexually dimorphic susceptibility to different types of stress, and has a possible role as a downstream mediator of PACAPergic stress response (Sragovich et al., 2019). The PACAP receptor, PAC1R, is a G-protein coupled receptor that undergoes alternative splicing of its mRNA transcript to diversify signaling pathways, with downstream responses in mediating neuroendocrine and cellular response to stress (Ajpru et al., 2002; Pilzer and Gozes, 2006). Splice variants of PAC1R are differentially expressed across tissue types and exert a differential effect on stress response and resiliency which appears to be age-dependent (Biran et al., 2020), further suggesting that variability in PAC1R expression mediates differential response to stress.

The gene encoding PAC1R (*ADCYAP1R1*) has several estrogen response elements (ERE), consensus sequences to which estrogen binds to regulate downstream transcription (Heldring et al., 2007). Effects of estrogen on PAC1 expression are mediated through estrogen receptor alpha (ERα) and subsequent binding to the ERE (Mercer et al., 2016). A single nucleotide polymorphism, rs2267735, located in a putative estrogen response element for the gene encoding ADCYAP1R1 predicts PTSD diagnosis in adult females, but not in males (Ressler et al., 2011). Presence of this risk allele leads to less efficient binding of estrogen to the ERE, and homozygosity for the risk allele in human females is associated with more PTSD symptoms and somatic anxiety symptom severity (Mercer et al., 2016; Ross et al., 2020). A recent longitudinal study in 8–13 year old children found that females homozygous for the risk allele had higher levels of fear-potentiated startle, an effect that was not observed in males (Jovanovic et al., 2020). Of note, the effect was observed on the second, but not first, visit of this longitudinal study, suggesting a possible effect of estrogen during pubertal development.

Prior studies in rodents have demonstrated estrous cycle-dependent *PACAP* mRNA expression in several brain regions, including in the paraventricular nucleus of the hypothalamus and in the pituitary gland (Moore et al., 2005). Complementing data from rodents suggest that increased estrogen levels and fear conditioning also induce *PAC1R* transcription, further suggesting that circulating hormone levels exert a sexually dimorphic response to stress, in part through PACAPergic signaling (Ressler et al., 2011; King et al., 2017).

Most human and animal studies on the effect of circulating gonadal hormones on fear processes have focused on the role of estrogen. In human studies of the impact of menstrual cycle on fear learning, most consistent data point to improved extinction recall with higher estradiol levels in healthy subjects (Milad et al., 2010; Li and Graham, 2016; Pineles et al., 2016). This finding is reversed in women with a diagnosis of PTSD, who show deficits in fear extinction during phases with highest estradiol (Pineles et al., 2016). Most animal studies of the effect of naturally-cycling endogenous estrogen on fear have also focused on extinction recall; however, methodological differences in handling and behavioral protocols complicate composite data interpretation. In 2001, Gupta et al. showed increased freezing and more rapid fear extinction during contextual fear conditioning in ovariectomized female rats compared to naturally cycling animals (Gupta et al., 2001). The study also showed impaired extinction of fear in males and ovariectomized females compared to sham operated females, suggesting that naturally cycling gonadal hormones enhance extinction learning in gonadally intact, healthy animals. Consistent with this finding, inhibition of estradiol synthesis impairs extinction recall in male rats (Graham and Milad, 2014).

In addition to the effects of estrogen, cyclic levels of progesterone can exert a change in transcription through the action of the progesterone receptor (Schumacher et al., 2014), the expression of which is regulated by circulating levels of estradiol, the primary endogenous estrogen (Quadros and Wagner, 2008). Fast-acting progesterone signaling is mediated through its metabolite, allopregnanolone, which acts as an allosteric modulator of γ-aminobutyric acid (GABA_A_) receptors and an antagonist at N-methyl-D-aspartate (NMDA) receptors, exerting an anxiolytic effect on behavior (for a review, see (Liang and Rasmusson, 2018)). Women with PTSD show a deficit in the conversion of progesterone to allopregnanolone, which is also associated with decreased extinction recall during high estrogen and progesterone phases (Pineles et al., 2016, Pineles et al., 2018, Rasmusson and Pineles, 2018).

Testosterone regulates genomic expression through its metabolites, dihydrotestosterone (DHT), which binds to the androgen receptor, and estradiol, which binds to the estrogen receptor (Durdiakova et al., 2011). Little is known about the role of the androgen receptor in the mediation of PTSD symptomatology. In a rodent model using predator scent stress to examine PTSD- and stress-related symptomatology, Fenchel and colleagues found that adult male rats with the greatest behavioral response had a downregulation of estrogen receptor alpha and androgen receptors in the hippocampus (Fenchel et al., 2015). These results suggest downstream transcriptional changes may be mediating PTSD symptomatology via the androgen receptor, however, these targets have not been identified to date.

A few clinical studies have examined the role of testosterone on differential gene expression after exposure to a traumatic stressor, with inconsistent results. Circulating serum levels of testosterone were found to be elevated in patients with PTSD, although this change was not observed in patients with comorbid MDD or alcohol dependence (Karlović et al., 2012). Another study found no change in plasma testosterone, but noted decreased testosterone in the cerebrospinal fluid of soldiers with PTSD (Mulchahey et al., 2001). One hypothesis for the inconsistency between studies is the involvement of both gonadal and adrenal hormones in stress reactivity, as well as the timing of trauma. In one study of soldiers in a war-zone, Josephs and colleagues found that lower pre-deployment levels of both saliva cortisol and testosterone were associated with increased risk of developing PTSD after a traumatic stressor (Josephs et al., 2017). Another study supported the effect of an underlying biological vulnerability, demonstrating a correlation of testosterone levels before stressor exposure on the development of PTSD symptomatology (Reijnen et al., 2015).

Combined, these studies suggest a mechanism by which naturally-cycling gonadal hormones can exert a sexually dimorphic transcriptional response to a stressor; however, further work on the role of downstream signaling and potential modulation of these signaling pathways is warranted. Notably, a recent analysis suggests that the progesterone/estrogen ratio, rather than individual hormone levels, may be a more useful biomarker for the study of PTSD symptomatology in both humans and rodent models (Seligowski et al., 2020).

## 5 Role of epigenetic modifications

Epigenetic changes, such as DNA methylation and histone acetylation, are associated with development of PTSD (Uddin et al., 2010; Wolf et al., 2018; the Traumatic Stress Brain Study GroupLogue et al., 2020; McCarthy et al., 2009). In addition to a direct effect on transcription through action on hormone response elements, signaling through steroid receptors has been shown to exert sex-specific changes on the epigenetic modifications in the central nervous system (Xu et al., 2014). Recent work in humans and mice demonstrated a correlation of increased serum estradiol levels with increased DNA methylation in civilian traumatized women enrolled in the Grady Trauma Project, suggesting dynamic hormone regulation of gene expression. The same study found an association between PTSD diagnosis and methylation of CpG sites on a gene encoding histone deacetylase 4 (*HDAC4).* Validation of this observation was suggested by finding, in mice, that increased *Hdac4* transcription was associated with low estrogen levels after fear conditioning in naturally cycling females (Maddox et al., 2018). These results suggest that circulating estrogen levels at the time of trauma may modulate subsequent fear responses, in part through effect of methylation changes on transcriptional regulation. In further support of this hypothesis, a neuroimaging study of Rwanda genocide survivors showed a correlation of sex-specific methylation changes at the *NGFI-A* gene, encoding nerve growth factor-induced protein A, with fewer intrusive memories in male, but not in female, subjects (Vukojevic et al., 2014).

In addition to changes in methylation, epigenetic editing through histone modifications has been implicated in sex-specific fear modulation. Recent work by Sase and colleagues identified sex-specific epigenetic regulation of Cyclin-dependent kinase 5 (CDK5), after previously demonstrating a role of targeted histone acetylation of this gene in fear-memory retrieval in male mice (Sase et al., 2019). Using targeted histone modifications to enhance or suppress *Cdk5* gene expression *in vivo* in the nucleus accumbens of male mice, Heller and colleague found that histone acetylation, associated with transcriptional activation, increases resilience to social stress (Heller et al., 2016). The group then applied targeted histone modifications in the hippocampus of male and female mice that underwent fear conditioning with foot shocks, and found an attenuated response in female, but not male, animals (Heller et al., 2016). *Cdk5* mRNA levels and histone acetylation were increased in the hippocampus of male mice 24 h after foot shocks, which was not observed in female mice or male controls. Interestingly, targeted histone-specific acetylation of the *Cdk5* promoter, which increased expression of *Cdk5,* decreased long-term memory in female, but not male mice. This was accompanied by a female-specific phosphorylation of tau protein, a downstream target of Cdk5, suggesting one potential mechanism for a sex-specific signaling cascade (Heller et al., 2016).

Another recent study found a sex-specific effect of a histone variant, H2A.Z on fear learning in female mice (Ramzan et al., 2020). A conditional deletion of H2A.Z in the hippocampus reduced fear potentiation following stress-enhanced fear learning paradigm in female, but not male, animals (Ramzan et al., 2020). Together, these studies highlight the relevance of sex-specific epigenetic changes in the regulation of fear-related circuitry. They also raise new questions about epigenetic changes with age, hormonal status at the time of trauma, and potential use as targets in drug development.

## 6 Conclusions and future directions

Recent data from large-scale RNA sequencing of human tissues (e.g., the Genotype-Tissue Expression (GTEx) project) suggest that over one third of genes examined exhibit a tissue-specific, sex-biased gene expression (Oliva et al., 2020). Increased heritability and prevalence of PTSD diagnosis in women, as well as differential effects of psychotropic medications during treatment in male vs female patients, highlight the need for predictive biomarkers and individualized treatment. To date, few large scale genomic and transcriptomic studies examined sex-specific differences in PTSD, and gene-expression studies that do consider sex stratification, do not consistently control for cell types.

In addition to large scale genetic studies in humans, there are multiple approaches to aid in the mechanistic study of genetic factors of sex differences using rodents. For example, the Four-Core-Genotypes model is a transgenic mouse in which the testis-determining gene, Sry, is moved from the Y chromosome to an autosomal chromosome, producing XX mice that have testes, and XY mice that develop ovaries. This animal model is useful to differentiate between sexual dimorphism produced by the presence of the sex chromosomes, or by circulating gonadal hormones (Burgoyne and Arnold, 2016). Another approach in animal models is to genetically or pharmacologically manipulate sex-steroid receptors or enzymes regulating hormone synthesis. In both cases, examining trauma and stress responses in such rodent models could help to further differentiate genetic from hormone-dependent molecular and behavioral responses.

Future studies should also consider the role of naturally-circulating hormone levels on fear response and symptom development, as some phenotypes are not evident until after puberty, highlighting importance of controlling for naturally-cycling menstrual phase, as well as role of elevated testosterone, on transcription. Because cyclic binding of estrogen to EREs induces a feedback loop by which estrogen regulates levels of estrogen receptors, use of exogenous sources of estrogen may result in supranormal levels and downregulate receptor levels, hindering further exploration of estrogen effects (Ter Horst et al., 2009). Where possible, naturally-cycling animals should be used for the study of transcriptional changes on affective disorders, although the full extent of estrogen's role on the function of the HPA axis and sexual dimorphism in response to stress is beyond the scope of this review (see (Solomon and Herman, 2009; Bangasser and Wicks, 2017)).

As noted in Fig. 1, multiple factors are likely interacting to increase the risk of PTSD in women. Men and women likely experience a greater proportion of distinct types of index traumas, with women being more likely to be victims of physical and sexual trauma and men being more likely to experience combat or be a witness to violence. In the United States, this difference is in part due to a historical ban on women in ground-level combat. This ban was lifted in 2013, and further research on mental health effects of combat trauma in women is likely forthcoming (MacGregor et al., 2020). Multiple studies also suggest a role for cyclic gonadal hormones in fear learning, which likely includes changes at the level of transcription and epigenetic modifications. Sex-specific epigenetic changes also play a role in the transmission of intergenerational effects of trauma, which may predispose offspring to subsequent development of PTSD (Yehuda and Lehrner, 2018).

In summary, recent and emerging work in the field of PTSD research suggests that multiple genomic mechanisms, including pathways involved in glucocorticoid signaling, HPA axis regulation, immune response, and epigenetic regulation, exhibit sex differences in signaling underlying the pathophysiology of this disease; however, current treatment options do not account for these underlying changes. Future work will help to identify specific targets for a more personalized and biologically-informed approach to the treatment of PTSD and other devastating stress-related conditions.

## CRediT authorship contribution statement

**Olga Y. Ponomareva:** Conceptualization, Methodology, Writing – original draft. **Kerry J. Ressler:** Conceptualization, Writing – review & editing.

## Declaration of competing interest

KJR has received consulting income from Alkermes and Bioxcel, and is on scientific advisory boards for Janssen, Verily, and Resilience Therapeutics. He has also received sponsored research support from Takeda and Brainsway. None of these are related to the work presented here.
